# Human Cataract Mutations in EPHA2 SAM Domain Alter Receptor Stability and Function

**DOI:** 10.1371/journal.pone.0036564

**Published:** 2012-05-03

**Authors:** Jeong Eun Park, Alexander I. Son, Rui Hua, Lianqing Wang, Xue Zhang, Renping Zhou

**Affiliations:** 1 Susan Lehman-Cullman Laboratory for Cancer Research, Department of Chemical Biology, Ernest Mario School of Pharmacy, Rutgers University, Piscataway, New Jersey, United States of America; 2 McKusick-Zhang Center for Genetic Medicine, State Key Laboratory of Medical Molecular Biology, Institute of Basic Medical Sciences, Chinese Academy of Medical Sciences, Peking Union Medical College, Beijing, China; University of South Florida College of Medicine, United States of America

## Abstract

The cellular and molecular mechanisms underlying the pathogenesis of cataracts leading to visual impairment remain poorly understood. In recent studies, several mutations in the cytoplasmic sterile-α-motif (SAM) domain of human EPHA2 on chromosome 1p36 have been associated with hereditary cataracts in several families. Here, we have investigated how these SAM domain mutations affect EPHA2 activity. We showed that the SAM domain mutations dramatically destabilized the EPHA2 protein in a proteasome-dependent pathway, as evidenced by the increase of EPHA2 receptor levels in the presence of the proteasome inhibitor MG132. In addition, the expression of wild-type EPHA2 promoted the migration of the mouse lens epithelial αTN4-1 cells in the absence of ligand stimulation, whereas the mutants exhibited significantly reduced activity. In contrast, stimulation of EPHA2 with its ligand ephrin-A5 eradicates the enhancement of cell migration accompanied by Akt activation. Taken together, our studies suggest that the SAM domain of the EPHA2 protein plays critical roles in enhancing the stability of EPHA2 by modulating the proteasome-dependent process. Furthermore, activation of Akt switches EPHA2 from promoting to inhibiting cell migration upon ephrin-A5 binding. Our results provide the first report of multiple EPHA2 cataract mutations contributing to the destabilization of the receptor and causing the loss of cell migration activity.

## Introduction

Cataract, the lens opacity disease, is the leading cause of blindness in the world, accounting for 48% of the cases [1]. Congenital cataract (CC) is one of the common causes of visual impairment in infants up to 25% [2]. Recent studies have examined the excess clustering of the disease in families with a high risk for cataract developments [3]. In addition, as much as 40% of early-onset cataracts may have a genetic basis [4]. Genetic studies have identified numerous underlying mutations including crystalline genes (*CRYAA*, *CRYAB*, *CRYBB1*, *CRYBB2*, *CRYBB3*, *CRYBA3/A1*, *CRYBA4*, *CRYGC*, *GRYGD*, and *CRYGS*) [5]–[13], connexin genes (*GJA3*, *GJA8*) [14], [15], and intermediate-filament-like factors (*VIM*) [16]. Recent genetic analyses revealed an additional novel pathway for cataract formation, mediated by mutations in the Eph receptor tyrosine kinase-type A2 (*EPHA2*) [17]–[20].

Human EPHA2 resides within the critical region on chromosome 1p36 that was previously defined in an Australian family with autosomal dominant total congenital cataracts [21], [22]. A recent study on the variations in the *EPHA2* receptor tyrosine kinase gene within this region has identified a missense mutation c.2842G>T which substitutes an amino-acid from glycine to tryptophan at codon 948 (GGG>TGG: p.G948W) for autosomal dominant posterior polar cataracts in Caucasians [20]. In addition, other recent findings identified missense [c.2819C>T (p.T940I) in a Chinese family], frameshift [c.2915_2916delTG (p.V972GfsX39) in a British family] and splicing (c.2826-9G>A in an Australian family) mutations in EPHA2 in three independent families developing CC from different ancestral groups [19]. All of these mutations are located in the cytoplasmic sterile-α-motif (SAM) domain at the C-terminus of EPHA2 [20], [23], [24], suggesting that the SAM domain of EPHA2 may have an important role in the regulation of EPHA2 function and lens development.

The SAM domain is a conserved protein module in many key regulatory proteins, scaffolding proteins, and transcription factors. Mutations in the SAM domain have been observed to cause several human diseases [19], [20], [25]–[34]. For example, SAM domain mutations in the *TP63* have been shown to affect SUMO-1-mediated regulation which would influence the protein stability causing ectodermal dysplasia syndromes [31], [32]. These defects are derived from increased *TP63* ubiquitination as a result of the SAM domain mutation [29]. The 12p13 *ETV6* (*TEL*: translocation ETS leukemia) SAM domain mutations block polymerization of ETV6-NTRK3 (EN) and transformation activity [26], [33]. The importance of this domain has led to numerous studies on the structure and stoichiometry of SAM domain complexes [19]. However, although SAM domains are capable of forming both homo- and hetero-oligomers *in vitro*, it remains unclear how SAM domains mediate protein interactions and what mechanisms regulate its association *in vitro* or *in vivo*. The presence of a conserved SAM domain within the cytoplasmic region of all Eph receptors indicates that it may play a role in regulating Eph receptor signaling. Since SAM domains facilitate protein-protein interactions [24] through homo- and hetero-oligomerization with other SAM domains, it is possible that EphA2 SAM domain mutations interfere with receptor oligomerization or clustering into higher-order complexes essential for physiologic signaling [23].

In the human genome, there are 14 Eph-coding genes (9 EPHAs and 5 EPHBs) and 8 ephrin ligand-coding genes (5 EFNAs and 3 EFNBs) [35]. Eph-related receptor tyrosine kinases (RTKs) have been implicated in the control of axon guidance, cell migration, angiogenesis, and patterning of the nervous system. Our previous studies showed that ephrin-A5 acts as a ligand for EphA2 in the lens, and the loss of ephrin-A5 function leads to cataracts in mice [36]. Additionally, ephrin-A5 interacts with the EphA2 receptor to regulate the adherens junction complex by enhancing recruitment of β-catenin to N-cadherin [36]. However, a molecular mechanism of EPHA2 signaling through the SAM domain that regulates lens development remains unknown. Here, we show that the EPHA2 SAM domain is required for protein stability and that the receptor utilizes both ligand-dependent and independent mechanisms to regulate lens epithelial cell biology.

## Results

### Mutations in the SAM domain of *EPHA2* gene reduce protein levels

**Figure 1:**
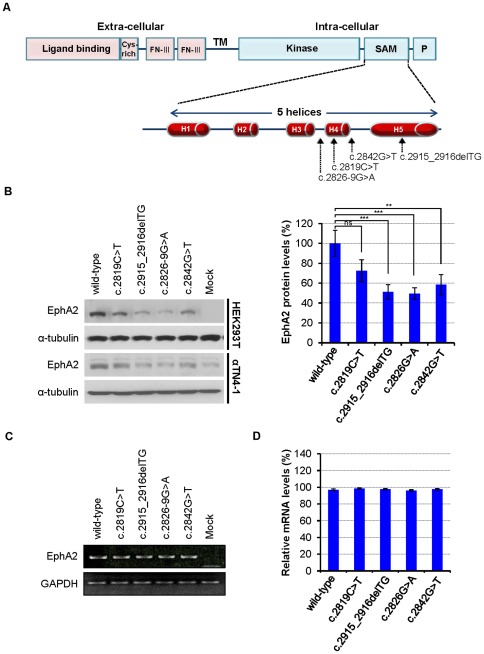
*EPHA2* cataract mutations in the SAM domain. (**A**) Schematic diagram showing the domains of EPHA2 receptor and the locations of four SAM domain mutations found in human cataracts (c.2819C>T; c.2915_2916delTG; c.2826-9G>A; and c.2842G>T) in the EPHA2 gene. FN-III: fibronectin type-III domain; TM: transmembrane domain; Kinase: protein tyrosine kinase domain; SAM: sterile-α-motif domain; P: PDZ-binding motif. The SAM domain comprises 5 α-hecices (H1–5). (**B**) Reduction of mutant EPHA2 protein levels in transfected cells expressing *EPHA2* mutants. Protein levels of *EPHA2* mutants are decreased in both HEK293T and αTN4-1 cells. The blot was reprobed with anti-α-tubulin as a loading control. The graphs represent the quantification of relative band intensity of EphA2 as connected by the levels of α-tubulin from three independent experiments. Total EphA2 protein band intensity was determined using ImageJ software. Mean values are presented with 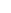
S.D as indicated. Statistical differences between multiple groups were analyzed using one-way analysis of variance (ANOVA). ***, *P*<0.001; **, *P*<0.01; *, *P*<0.05; and ns, not significant. Values of *P*<0.05 were considered to be statistically significant. (**C, D**) No difference between wild-type and mutant *EPHA2* genes in transcription levels. (**C**) Semi-quantitative RT-PCR and (**D**) Real-time PCR for wild-type and mutant *EPHA2* genes were performed using total RNA, isolated from transfected HEK293T cells. GAPDH transcript levels are used as controls. The graphs represent the quantification of western blots from three independent experiments.

Our previous observations on the role of the ephrinA5/EphA2 molecules on lens development [36] suggest that EphA2 may act as a critical mediator in lens function. Consistent with our hypothesis, it has been shown that mutations in the *EPHA2* gene within human chromosome 1p36 region lead to cataracts [17]–[20], [37]. Interestingly, four of the known mutations within *EPHA2* are located in the SAM domain of the C-terminal region of EPHA2 (Figure 1A) that serves as a potential protein interaction site [19], [20], [23], [24]. To examine the consequences of these mutations, we generated four mutant *EPHA2* genes: the missense mutants c.2819C>T (p.T940I) and c.2842G>T (p.G948W), the frameshift mutant c.2915_2916delTG (p.V972GfsX39), and the splicing mutant c.2826-9G>A (Figure 1A). In the c.2819C>T EPHA2 mutant, isoleucine replaces the wild-type threonine at residue 940 between H-3 and H-4 segments in the SAM domain [19]. The missense mutant c.2842G>T has a G
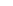
T mutation of codon 948 (GGG>TGG) resulting in the missense substitution of glycine by tryptophan [20]. The c.2915_2916delTG mutant has a deletion of 2 bp in exon 17 resulting in a mutant EPHA2 protein with a novel C-terminal polypeptide of 39 amino acid residues. The c.2826-9G>A substitution creates a novel splice acceptor site which adds an intronic sequence into the mRNA generating a novel 71 amino acid residues at the C-terminus, of which the last 39 residues are identical to that of the novel polypeptide produced by the c.2915_2916delTG frameshift mutation [19].

To investigate whether the EPHA2 SAM domain mutations affect EPHA2 expression, we examined EPHA2 protein levels. Wild-type and mutant *EPHA2* genes were transfected into HEK293T and mouse lens epithelial αTN4-1 cells. Wild-type EPHA2 is expressed at high levels in both HEK293T and αTN4-1 cells, while the mutant EPHA2 genes, c.2915_2916delTG, c.2826G>A and c. 2842G>T, showed low levels compared to the wild-type (Figure 1B). However, one of the mutant proteins, c.2819C>T, did not show a significantly lower level of expression from that of the wild-type protein, although it appeared to be somewhat reduced. We next examined whether these differences were due to the differences in transcription. Semi-quantitative (Figure 1C) and real-time RT-PCR (Figure 1D) reactions were used to investigate the mRNA levels. After transfection into HEK293T cells, PCR reactions for wild-type and mutant *EPHA2* genes were performed using total RNA of the transfected cells, and *EPHA2* PCR products were normalized to GAPDH transcript levels. No differences were found in mRNA level between the wild-type and mutants (Figure 1C,D), suggesting that these SAM domain mutations affect EPHA2 protein levels posttranscriptionally. We also expressed the wild-type and mutant EPHA2 SAM domain constructs as GST-fusion proteins in *E. coli* and found that the solubility of the mutant proteins was significantly reduced (Figure S1A,B) indicating an alteration of protein conformation. These results together suggest that mutations in the SAM domain of *EPHA2* receptor reduce protein stability, and the reduced mutant protein levels may also in part, be a result of defective protein synthesis or maturation related to their altered conformation.

**Figure 2:**
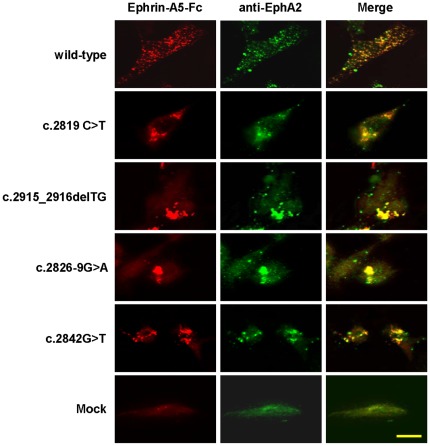
Subcellular localization of the wild-type and mutant *EPHA2* receptors on transfected EphA2^−/−^ MEF (E13.5) cells. EphA2 knockout MEF cells expressing wild-type or mutant *EPHA2* were incubated with clustered ephrin-A5-Fc before fixation and treated with anti-Fc antibodies (red). After washing with PBS, cells were counterstained with anti-EphA2 antibody (green). Images were captured using a Nikon Eclipse C1 confocal microscope. Scale bar, 50 µm.

We also investigated EPHA2 protein expression using ligand-mediated immunofluore- scence staining. Detectable binding of ephrin-A5 was observed in EphA2^−/−^ MEF cells expressing wild-type and mutant *EPHA2* genes (Figure 2). Wild-type EPHA2 showed evenly distributed small protein aggregates throughout the cells (Figure 2). In contrast, EPHA2 mutants exhibited large protein aggregates, suggesting that the mutations in the SAM domain lead to enhanced protein aggregation, consistent with decreased GST-SAM domain fusion protein solubility in *E. coli* (Figure S1B).

### The ubiquitin-proteasome pathway mediates degradation of EPHA2 mutants

**Figure 3:**
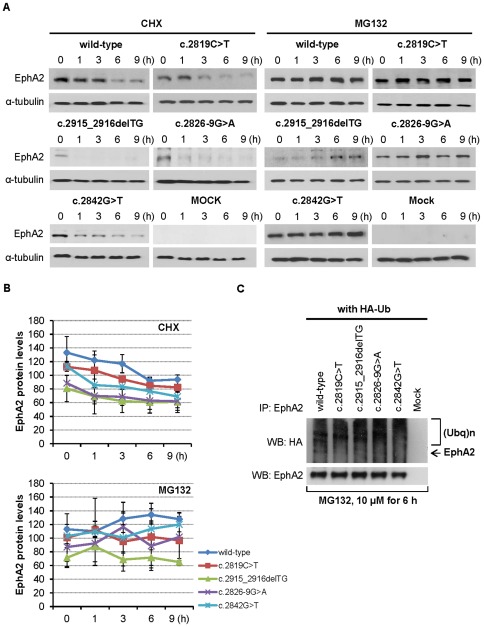
EPHA2 degradation is mediated by proteasomal pathway. (**A**) Mutant EPHA2 proteins have a reduced half-life. HEK293T cells were treated for indicated time with the protein biosynthesis inhibitor CHX (50 µg/mL) or the proteasome inhibitor MG132 (10 µM). Cell lysates were immunoblotted with anti-EphA2 antibody. Lysates were resolved by SDS-PAGE and western blot analysis was performed using indicated antibodies as described in the Materials and Methods. The blot was reprobed with anti-α-tubulin as a loading control. (**B**) Quantification of EphA2 protein levels over time. Mean values are presented with 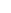
S.D as indicated. (**C**) EPHA2 mutants have increased ubiquitination. Cells transfected with *EPHA2* and HA-tagged ubiquitin were treated with 10 µM MG132 for 6 hours, and were then lysed. Immunoprecipitated EphA2 was further analyzed with western blotting using anti-HA antibodies to detect ubiquitinated EphA2 as described in the Materials and Methods. The smear band is characteristic ubiquitin immunoreactivity. The amount of total EphA2 is shown as a loading control.

To determine whether the SAM domain mutations reduce the half-life of EPHA2 protein, we examined effects of cycloheximide (CHX), a protein synthesis inhibitor. HEK293T cells expressing wild-type and mutant *EPHA2* genes were treated with 50 µg/mL CHX to block new protein synthesis. EPHA2 proteins with mutations in the SAM domain showed more rapid degradation kinetics compared to the wild-type (Figure 3A). The half-life of wild-type EPHA2 is approximately 3 hours (Figure 3B), whereas the half-life of the mutant proteins has been reduced to less than 1 hour (Figure 3A,B). These results demonstrate that mutations in the SAM domain result in rapid EPHA2 proteolysis.

To investigate whether EPHA2 degradation is mediated through proteasomal and lysosomal pathways, cells were treated with either the specific proteasome inhibitor MG132 or the lysosomal proton pump inhibitor bafilomycin A1. In the presence of 10 µM MG132, the expression levels of mutant EPHA2 proteins gradually increased over time (Figure 3A,B). Co-treatment with CHX and MG132 also largely prevented degradation of EPHA2 proteins (Figure S2A,B). In contrast, bafilomycin A1 did not affect the levels of EPHA2 proteins (Figure S3). These results indicate that EPHA2 proteins are degraded by proteasomes rather than lysosomes, and that the SAM domain is critical in modulating the degradation.

Proteasomal inhibition is normally associated with the accumulation of polyubiquitinylation on proteins. To confirm EPHA2 is degraded via the ubiquitin-mediated proteasomal pathway, wild-type and mutant *EPHA2* genes were cotransfected with HA-tagged ubiquitin (HA-Ub) in the presense of MG132. The EPHA2 proteins were then immunoprecipitated with anti-EphA2 antibody and analyzed with western blotting for the presence of ubiquitin using an anti-HA antibody. The anti-HA antibody detected an increase in the intensity of high-molecular-mass EPHA2 proteins (Figure 3C), suggesting that the mutant proteins had increased polyubiquitination. Taken together, our data indicate that these particular mutations in the SAM domain of the *EPHA2* receptor enhance proteasome-mediated EPHA2 protein degradation.

### Ephrin-A5 stimulation induces tyrosine phosphorylation of EPHA2 mutants

**Figure 4:**
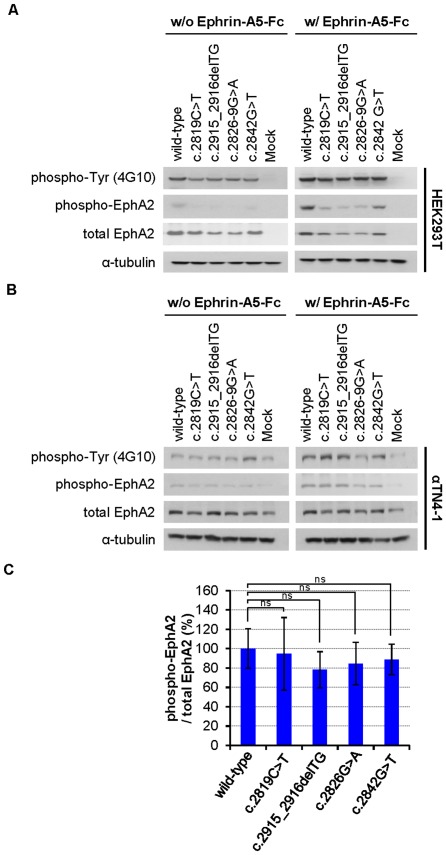
Tyrosine phosphorylation of EPHA2 receptor by ephrin-A5 is not affected by SAM domain mutations. (**A, B**) Ephrin-A5 ligand stimulates EPHA2 phosphorylation. HEK293T (**A**) and αTN4-1 (**B**) cells were grown to confluence and growth factor-starved for 24 hours. 2 µg/mL cross-linked ephrin-A5-Fc was then added to the starvation media and cell lysates were immunoblotted with indicated antibodies. Western blot analysis was performed as described in the Materials and Methods. The blot was reprobed with anti-α-tubulin as a loading control. (**C**) The ratios of levels of phospho-EphA2 to total EphA2 are similar between the wild-type and mutant EPHA2 proteins. The graphs show total band intensity of anti-phospho-EphA2 immunoblot to total EphA2 and represent the average of three independent experiments. Quantification of phospho-EphA2 protein/total EphA2 protein levels was performed using ImageJ software. Mean values are presented with 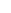
S.D as indicated. Statistical differences between multiple groups were analyzed using one-way analysis of variance (ANOVA). Values of *P*<0.05 were considered to be statistically significant. ns: No statistically significant difference between the two groups.

One important question is whether the EPHA2 mutants can still be activated by ephrin-A5. To address this question, *EPHA2* wild-type and mutant genes were transfected into HEK293T and αTN4-1 cells, and the cells were stimulated with clustered recombinant ephrin-A5-Fc at 37°C for 30 minutes. The cell lysates were analysed with two phospho-specific antibodies: anti-phospho-Tyrosine (4G10) and anti-phospho-EphA2. EPHA2 wild-type and mutants showed similar tyrosine phosphorylation in response of ephrin-A5 ligand (Figure 4). This analysis showed that all mutants were autophosphorylated, although the levels were lower than the wild-type due to reduced protein concentration (Figure 4A,B). Ephrin-A5 stimulation further enhanced EPHA2 tyrosine phosphorylation. To confirm that EPHA2 is specifically phosphorylated, we probed the cell lysates with an anti-phospho-EphA2 (Tyr594) antibody, which detects transfected levels of EPHA2 proteins only when phosphorylated on Tyr594 and does not cross-reacted with other activated protein tyrosine kinases [38]. As shown in Figure 4A and Figure 4B, ephrin-A5 also enhanced EPHA2 Y594 phosphorylation in both HEK293T and αTN4-1. Quantification of intensity of the phosphorylated EPHA2 after protein level corrections showed similar activity between the wild-type and mutant EPHA2 proteins, although the mutants had reduced protein expression level (Figure 4C).

### *EPHA2* cataract mutations result in the loss of the ability to promote cell migration

**Figure 5:**
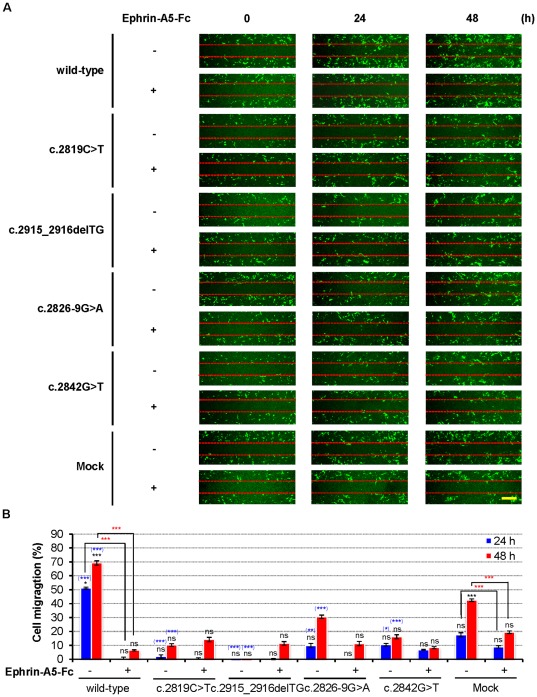
SAM domain of *EPHA2* is essential for ligand-independent promotion of cell migration. (**A**) Mutant *EPHA2* genes fail to promote αTN4-1 cell migration. αTN4-1 cells were grown to confluency and serum-starved for 24 hours. A scratch wound was made with a micropipette tip and the edge of cells was marked. 2 µg/mL cross-linked ephrin-A5-Fc was then added to the starvation media, and cells were allowed to migrate toward the center of the wound and photographed at the indicated times (representative figure of three independent experiments). The position of the initial scratch is indicated by dotted lines. Scale bar, 500 µm. (**B**) Quantification of the effects of *EPHA2* genes on αTN4-1 cell migration. The graphs represent the measurement of migration distance from three independent experiments. Mean values are presented with 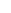
S.D as indicated. Statistical differences were analyzed using one-way analysis of variance (ANOVA) or calculated by a two-tailed student t-test. ***Black asterisks*** **,** comparison between time 0 and 24 hours and time 0 and 48 hours; ***Blue asterisks*** **,** comparison between the mock groups and the listed wild-type or mutant *EPHA2* genes at 24 or 48 hours; ***Red asterisks*** **,** comparison between untreated and treated conditions at 24 or 48 hours. ***, *P*<0.001; **, *P*<0.01; *, *P*<0.05; and ns, not significant. Values of *P*<0.05 were considered to be statistically significant.

To examine how the cataract mutations affect EPHA2 function, we investigated EPHA2 regulation on cell migration using the wound-healing assay, a common method in analyzing cell migration. A confluent cell monolayer was wounded using a pipette tip, introducing a cell-free area, and the migration of cells into the wound was monitored by capturing images at the indicated time points. In the absence of ephrin-A5 stimulation, wild-type EPHA2 promoted cell migration, while *EPHA2* mutations in the SAM domain greatly reduced this ability (Figure 5A). Cells expressing wild-type *EPHA2* began to migrate into the wound at 24 hours, while ephrin-A5 treatment significantly impaired the EPHA2-induced migration. Wound-healing assays were also quantified by measuring the distance by which transfected cells migrated into the cell-free region. Cells expressing wild-type EPHA2 migrated markedly faster than the mutants, occupying 51.25% (3.075 mm/6 mm) of the cell-free area after 24 hours and to 69.17% (4.15 mm/6 mm) after 48 hours (Figure 5B). The cataract mutations reduced the ability of EPHA2 to promote cell migration (Figure 5A,B). Although the wild-type EPHA2 promoted cell migration, treatment with ephrin-A5 impaired EPHA2-mediated cell migration (Figure 5A,B). Similar results were obtained using HEK293A cells (Figure S4A,B). In addition, statistical analysis showed there were significant differences between wild-type EPHA2 and the mock control groups (Figure 5B and Figure S4). The effects of mutant *EPHA2* genes were also statistically different compared to that of mock-transfected αTN4-1 cells (Figure 5B).

These observations demonstrate that EPHA2 promotes cell migration in the absence of ligand. However, ligand stimulation resulted in a switch of *EPHA2* function, turning promotion to inhibition of cell migration.

### EPHA2 SAM domain mutations reduce Akt activation

**Figure 6:**
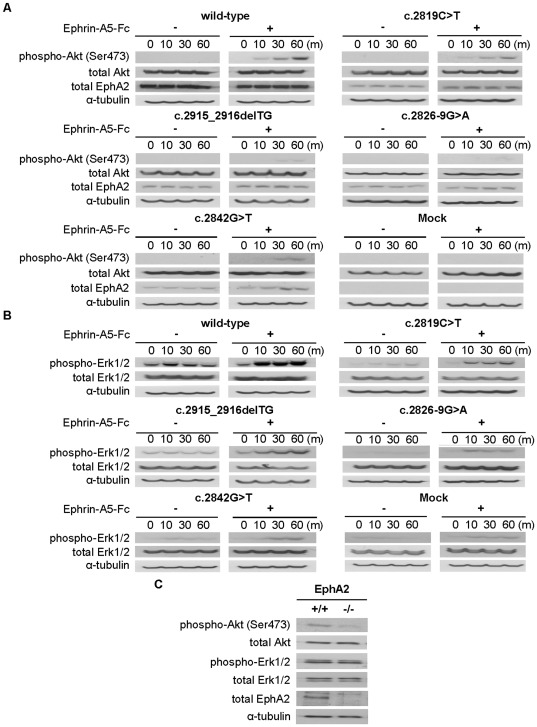
Ligand-stimulated EPHA2 activation regulates Akt and Erk activation. (**A, B**) Mutant EPHA2 proteins exhibit reduced activation of Akt and Erk by ephrin-A5. HEK293T cells were grown to confluence and serum-starved for 24 hours. 2 µg/mL cross-linked ephrin-A5-Fc was then added to the starvation media and cell lysates were immunoblotted with anti-phospho-Akt (Ser473) or anti-phospho-Erk (1/2), and then reprobed with anti-α-tubulin as a loading control. (**C**) Inactivation of EphA2 gene leads to reduction of Akt activity in mouse lenses. Each lens was prepared from 22 days old mice and extracted with lysis buffer. Total lens proteins were resolved by SDS-PAGE and western blot analysis was performed using indicated antibodies as described in the Materials and Methods. The blot was reprobed with anti-α-tubulin as a loading control.

To assess whether the SAM domain mutations affect EPHA2 biochemical functions, we determined the effects of EPHA2 on Akt and extracellular signal-regulated kinase (Erk) 1/2 signaling [18], [39], [40]. HEK293T cells transfected with wild-type and mutant *EPHA2* genes were serum-starved for 24 hours, then treated with 2 µg/mL ephrin-A5-Fc at the indicated time points. Stimulated cells were lysed and examined for Akt and Erk activation. Ephrin-A5 stimulation of HEK293T cells expressing wild-type EPHA2 resulted in an increase of Akt phosphorylation at Ser473 in a time-dependent manner. In contrast, ephrin-A5-induced Akt phosphorylation was severely reduced in *EPHA2* mutant-transfected cells after ephrin-A5 stimulation (Figure 6A). Akt phosphorylation was almost undetectable in cells expressing c.2915_2916delTG, c.2826-9G>A and c.2842G>T. Similar results were obtained using αTN4-1 cells (Figure S5A,B). The ability of the various EphA2 genes to activate Akt closely correlated with the receptor protein levels, as evidenced by the similar ratios of phospho-Akt (Ser473) to total EphA2 protein signals (data not shown).

To determine whether the SAM domain mutations affect p44/p42 mitogen-activated protein kinase (MAPK) phosphorylation, lysates of HEK293T cell transfected with various *EPHA2* genes were probed with anti-phospho-Thr202/Tyr204 Erk1/2 antibody. Ephrin-A5 stimulation of HEK293T cells expressing wild-type EPHA2 resulted in a robust increase in Erk phosphorylation, while cells expressing the *EPHA2* mutant genes showed little or no activation (Figure 6B). In addition, the intensity of the phosphorylated Erk1/2 showed also a dependence on EPHA2 protein expression levels, similar to Akt activity (data not shown).

To examine whether EphA2 inactivation affects activation of Akt and Erk1/2 *in vivo*, we analyzed the lenses of postnatal day 22 (P22) EphA2^+/+^ and EphA2^−/−^ mice. Akt activation was clearly detected in the wild-type EphA2 mouse lens (Figure 6C). However, Akt phosphorylation was almost undetectable in EphA2^−/−^ mice. In contrast, levels of phospho-Erk1/2 did not show any decrease (Figure 6C).

## Discussion

*EPHA2* is a member of the Eph family receptor tyrosine kinases, and is strongly expressed in the cortical lens fiber cells. Recent studies have shown that mutations or deletion of EphA2 gene lead to cataracts in humans and mice [18]–[20]. Complementing these observations, our previous study identified that the loss of ephrin-A5 also leads to cataracts in mice [36], indicating that EphA2 serves as a receptor for the ligand in maintaining the clarity of the crystalline lens. The current study aims to define the nature of the *EPHA2* SAM domain mutations. Previous studies showed that the SAM domains in Eph receptors may have multiple functions [38], [41]–[46]. The SAM domain of EphB2 receptor can self-associate and forms oligomers [42], [44], although the exact function is not known at present. In contrast, the SAM domain of EphA4 receptor has been shown to negatively regulate receptor kinase activity [43], but is not required for topographic mapping in the brain [41]. This domain has also been shown to mediate recruitment of downstream signaling molecules [45], [46]. A recent study by Fang et al. showed that the tyrosine 929 in the SAM domain of EphA2 is required for Ephrin-A1-induced vascular assembly [38]. Our analysis revealed an additional function in the maintenance of receptor stability.

### *EPHA2* SAM domain mutations cause increased receptor proteolysis

Eph receptors have an extracellular region consisting of an ephrin-binding domain and two fibronecin type III repeats, and an intracellular region comprised of a regulatory juxtamembrane domain, a tyrosine kinase domain, a SAM domain and a PDZ-binding motif [23]. SAM domains mediate important protein-protein interactions and are found in a variety of signaling molecules that exert diverse cellular functions [25]. A highly conserved SAM domain in the cytoplasmic region of all Eph receptors is located at the C-terminal region of the receptors and contain five α helical domains [25], [47]. Our studies suggest that the SAM domain of the EPHA2 protein modulates ubiquitinylation and regulates the stability of the receptor. As a first step to examine the specific effects of *EPHA2* SAM domain mutations, we investigated the stability of the mutant EPHA2 proteins in two different cell lines, HEK293T and αTN4-1. Our results show that the EPHA2 proteins with SAM domain mutations exhibit increased degradation in a proteasome-dependent pathway, as treatment with the proteasome inhibitor MG132 enhanced EPHA2 protein levels. Ubiquitin-mediated proteasomal degradation pathway plays an important role in regulating a wide variety of cellular processes, since many proteins are degraded through this pathway [48], [49]. Thus we conclude that human EPHA2 SAM domain mutations cause cataracts through the reduction of EPHA2 protein levels.

Although the SAM domain modulates EPHA2 stability and protein solubility, it cannot be ruled out that the PDZ-binding motif may also contribute to EPHA2 stability, since the two mutants without the PDZ-binding domain, c.2915_2916delTG and c.2826-9G>A, showed lower steady state protein levels.

### Mutations in the SAM domain affect EPHA2 protein solubility and subcellular localization

Many human diseases such as Alzheimer's and Parkinson's diseases as well as cataracts, have been demonstrated as “protein condensation diseases”, in which the pathogenic proteins form insoluble aggregates [50]. It has been shown also previously that the change of sequence parameters by mutations can affect protein expression and solubility *in vivo* [50]. To gain insights into the effects of the cataract mutations on protein solubility, we generated SAM domain fusion proteins from the wild-type and mutant *EPHA2* receptors. Interestingly, all the GST-SAM domain mutant fusion proteins were insoluble in contrast to the wild-type GST-SAM domain fusion protein. These results indicate that SAM domain mutations influence protein stability and degradation rate through changes of solubility and folding efficiency. Several pathogenic proteins have been reported to have mutations in specific domains which induce aggregate formation and deplete the proteins from their normal cellular environment due to incorrect protein folding [51], [52]. Indeed we also observed that the mutations in the SAM domain of *EPHA2* alter the patterns of subcellular distribution. The mutant proteins form much bigger aggregates than the wild-type. These observations support the notion that the *EPHA2* cataract mutations induce protein misfolding leading to instability, which may lead to cellular disorganization and eventual lens opacity. Recent studies have shown that the solubility of several cataract-linked mutants of human γD-crystallin is severely compromised as a result of the mutations [50]. Well known examples are the R36S and the P23T mutants of human γD-crystallin, which can spontaneously crystallized at very low concentrations *in vitro* as a result of the lowered solubility of the mutant protein [50]. Similarly, mutant *EPHA2* receptors form large protein aggregates, consistent with an insolubility issue.

### EPHA2 regulates cell migration

It has been well established that wild-type EphA2 regulates cell migration, proliferation and invasion in a number of cell types [53]–[59]. Given the wealth of information linking EphA2 to cell migration, we evaluated the functional effects of the EPHA2 SAM domain mutations using the wound-healing assay. We showed that EPHA2 promotes cell migration in the absence of ligand stimulation, and that the SAM domain mutations diminish this activity, possibly due to the reduction of EPHA2 protein levels. Statistical analysis showed that there were significant differences between the wild-type and mutant *EPHA2* genes. Similar enhancement effects in cell migration were reported following EphA2 expression in other cell types, including MDA-MB-231 breast cancer cells [59], PC3 carcinoma cells [60], U373 glioblastoma cells, and U87 glioblastoma cells [57]. Inactivation of EphA2 has also been shown to impair cell migration [58], [61]. For example, EphA2-deficient murine pulmonary microvascular (MPMEC) endothelial cells have impaired angiogenesis [58], [61]. Ligand-independent promotion of cell migration by EphA2 is likely mediated through interaction with Ephexin4 and the eventual activation of Rac1 [59].

Ephrin ligand binding induces Eph receptor phosphorylation and activation [53], [56], [57], [61]. The activation of EphA2 has been shown to negatively regulate cell migration [57], [60], [62], proliferation [53] and invasion [54]–[56] in a number of cell lines including U373, U87, A172, PC3, MDA-MB-231, MDA-MB-435, MCF10A, Capan2, G48a, U87 and U251. We also observed that stimulation of EPHA2 with ephrin-A5 resulted in the inhibition of migration of HEK293T and αTN4-1 cells. Although EphA2 activation inhibits cell migration in these studies, the opposite effects have also been reported [58], [61], [63]. Brantley-Sieders et al., showed that stimulation of lung microvascular endothelial cells (MPMEC) and bovine pulmonary microvascular endothelial cells (BPMEC) with ephrin-A1 induces cell migration [58], [61]. In human cardiac stem cells (CSC), ephrin-A1 promotes cell migration and enhances cardiac repair [63]. Taken together, our results are consistent with previous studies showing that EPHA2 is a major regulator of cell migration, and that the effects depend on whether the ligand is present and the cellular context.

### Akt activation serves as a switch for EPHA2 function in cell migration

Mechanism underlying EPHA2 suppression of cell migration after ligand stimulation remains incompletely defined. The phosphoinositide 3-kinase (PI3K) signaling pathway has been shown to regulate cell growth, proliferation and migration [62], [64]. Akt and Akt-related serine-threonine kinases are activated by ligand stimulated growth factor receptor signaling in a PI3K-dependent manner [64]. We have shown here that activation of EPHA2 by ephrin-A5 in both αTN4-1 and HEK293 cells resulted in Akt activation, which correlates with inhibition of cell migration. Akt activation has been shown to inhibit migration and invasion of MDA-MB-231, MDA-MB-435 and SUM-159-PT breast cancer cells [65]. Another recent study has shown that down-regulation of Akt1 enhanced epidermal growth factor (EGF)-stimulated cell migration in MCF-10A breast cancer cells [66]. These results are consistent with our observation that activation of Akt mediates the inhibitory effects on migration. Thus, Akt activation serves as molecular switch for EphA2 function in cell migration.

The effects of EphA2 activation on Akt activity can vary depending on the cellular context. A number of studies have shown that stimulation of EphA2 by ephrin-A1 resulted in inhibition of Akt activity in certain cell lines (U87, U251, A172, MCF7, G48a, ES2, HEYA8 and corneal epithelial cells) [39], [54], [57], [62]. However, Akt is activated following ephrin-A1 stimulation in a number of other cell lines (B16, LNCaP, BxPC-3, PANC-1 and MIA PaCa-2) [39], [67]. Consistent with these latter studies, we observed Akt activation by ephrin-A5 stimulation, and found that Akt (Ser473) became highly phosphorylated in response to the addition of the ligand. The molecular mechanisms underlying this activation need further investigation. We also showed Akt activation depends on EphA2 expression levels, and reduced Akt activation may be responsible at least in part for cataract development in patients with EphA2 SAM domain mutations, since our *in vivo* studies reveal that Akt activation was significantly reduced in EphA2 knockout mouse lens. Thus, the ability of EPHA2 to cause Akt dephosphorylation or phosphorylation appears to depend on cell specific environment, and there may be kinase-dependent and -independent pathways that regulate Akt activity.

In sum, our studies show that mutations in the SAM domain of *EPHA2* receptor induce EPHA2 protein instability. In addition, ephrin-A5 stimulation induces Akt activation, which in turn suppresses cell migration. These observations provide new insights in the mechanism by which defects in to *EPHA2* signaling causes human cataracts.

## Materials and Methods

### Ethics statement

Animal studies were performed under standard conditions and treated in accordance with the Guidelines for the Animal Care and Use Committee at Rutgers University and ARVO Statement for the Use of Animals in Opthalmic and Vision Research (Rutgers approval ID number #93-052).

### Mice and tissue extraction

The EphA2^−/−^ [68] mice were kindly provided by Dr. Bingcheng Wang (Case Western Reserve University School of Medicine, Cleveland, Ohio, USA). All animals used were 22 days old in this study. For tissue extraction, lenses were dissected from mouse eyes and homogenized in ice-cold lysis buffer containing 50 mM Tris-HCl (pH 8.0), 150 mM NaCl, 1% NP-40, 100 µg/mL phenylmethylsulfonyl fluoride (PMSF), 1 µg/mL aprotinin, 10 µg/mL leupeptin, and 1 mM Na_3_VO_4_. The samples were cleared by centrifugation at 13,000 g for 2 minutes and used in western blot analysis.

### Cell culture and transfection

Human embryonic kidney 293T (HEK293T) cells obtained from American Type Culture Collection (ATCC), and mouse lens epithelial αTN4-1 cells [69] were generously provided by Dr. Bingcheng Wang (Case Western Reserve University School of Medicine, Cleveland, Ohio, USA). Cells were maintained in Dulbecco's modified Eagle's medium (DMEM, Sigma-Aldrich, USA) containing 4500 mg/L glucose/L, 584 mg/L L-glutamine/L with 10% fetal bovine serum (FBS) and 1% Penicillin-Streptomycin solution (10000 units penicillin and 10 mg streptomycin/mL in 0.9% NaCl, Sigma-Aldrich, USA) at 37°C. Primary mouse embryonic fibroblasts (MEFs) were isolated from EphA2^−/−^ E13.5 embryos and cultured in DMEM supplemented with 10% FBS [70]. Cells from passage 2 were used for transfection. Transient transfection was performed using Lipofectamine 2000 (Invitrogen, Carlsbad, CA, USA) following the manufacturer's instructions. Protein levels were evaluated by immunoblotting 2 days after transfection.

### Expression of *EPHA2* genes

The human *EPHA2* wild-type (GenBank NM_004431.3) and c.2842G>T were cloned using PCR previously [19]. Three other mutant cDNAs (c.2819C>T; c.2915_2916delTG; c.2826-9G>A) were generated by DNA synthesis (GeneScript USA Inc.). All cDNAs were cloned into the eukaryotic expression vector pcDNA3.1 (Invitrogen, Carlsbad, CA, USA). All mutations were verified by DNA sequencing.

### Antibodies and reagents

Antibodies used for immunoblot are from the following sources: anti-phospho-EphA2 (Tyr594) (1∶500, #3970), anti-Akt (1∶1000, #4691S), anti-phospho-Akt (Ser473) (1∶1000, #9271S), anti-Erk1/2 (1∶1000, #4695S), anti-phospho-Erk1/2 (1∶1000, #9101S) and anti-HA-Tag (1∶1000, #2367) from Cell Signaling Technology (Beverly, MA, USA); anti-EphA2 (1∶500, #E1026), anti-α-tubulin (1∶5000, #T6074), cycloheximide (CHX) (#C4859) and MG132 (Z-Leu-Leu-Leu-al) (#C2211) from Sigma-Aldrich (USA); bafilomycin A1 (#ab120497) and anti-EphA2 (1∶1000, #ab5386) from Abcam (Cambridge, MA) and anti-phospho-tyrosine (4G10) (1∶3000, #16-316) from Milipore Corporation (Billerica, MA).

### Ephrin-A5 preparation and treatment

Recombinant ephrin-A5-Fc protein was purchased from R&D Systems (Minneapolis, MN, USA; #374-EA). To form clustered ephrin-A5, ephrin-A5-Fc (2 µg/mL) was cross-linked with anti-human Fc IgG (Jackson Immuno-Research, Immuno-Research, West Grove, PA, USA) at a 5∶1 ratio in µgs for 2 hours at 37°C as described in our previous studies [71]. Transfected cells were stimulated by ephrin-A5-Fc after serum starvation at 37°C overnight.

### Western blot analysis

Tissue and cells were lysed in lysis buffer containing 50 mM Tris-HCl (pH 8.0), 150 mM NaCl, 1% NP-40, 100 µg/mL phenylmethylsulfonyl fluoride (PMSF), 1 µg/mL aprotinin, 10 µg/mL leupeptin, and 1 mM Na_3_VO_4_ for 30 minutes at 4°C. Lysates were cleared by centrifugation at 13,000 g for 2 minutes. 30 µg of protein samples were boiled in 2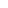 SDS-PAGE loading buffer and fractionated on 7.5% (w/v) SDS-PAGE gels and transferred to nitrocellulose membranes (Bio-Rad, Hercules, CA, USA). After blocking with 5% (w/v) dried skim milk in PBST (PBS with 0.1% Tween 20) for 1 hour, the membranes were probed with the indicated antibodies, coupled with a HRP-conjugated secondary antibody. Bands were visualized with chemiluminescence using ECL western blotting detection reagents (Amersham Pharmacia Biotech, UK) according to the manufacturer's instructions. Protein band intensities were quantified using NIH ImageJ software.

### Immunoprecipitation

Briefly, various antibodies were added to the cell lysate, and incubated at 4°C overnight. The immunocomplex was recovered by using protein A-agarose beads (Roche Molecular Biochemicals, Indianapolis, IN, USA; #1719408) and centrifugation. After washing the protein A-Agarose beads five times with lysis buffer, the precipitated proteins were recovered by boiling in 40 µL 2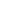 SDS-PAGE loading buffer for 5 minutes.

### Detection of ubiquitination

HEK293T cells at 80% confluence were co-transfected with 2 µg of *EPHA2* plasmid DNA and 1 µg of HA-tagged ubiquitin (HA-Ub) using the Lipofectamine 2000 (Invitrogen, Carlsbad, CA, USA). At 48 hours post-transfection, cells were treated with 10 µM MG132 for 6 hours, and then were lysed with cell lysis Buffer. Cell lysates were clarified at 13,000 g for 2 minutes. Immunoprecipitation was carried out using an EphA2 antibody at 1 µg/mg of total protein at 4°C for 2 hours. Immune complexes were collected using protein A agarose (Millipore, Billerica, MA, USA) at 4°C for 1 hour. The beads were then washed with immunoprecipitation wash buffer containing 20 mM Tris-HCl (pH 7.4), 10% glycerol, 50 mM NaCl, 0.2% NP-40, 0.5 mM PMSF and 0.5 mM Na_3_VO_4_. The samples were re-suspended in SDS sample buffer and fractionated on 7.5% polyacrylamide gel. Immunoprecipitates or total cell lysates were analysed by western blotting as described above and probed with the anti-HA-Tag antibody.

### Inhibition of protein synthesis and degradation

HEK293T cells were transfected with the *EPHA2* wild-type and four mutants, c.2819C>T, c.2915_2916delTG, c.2826-9G>A, and c.2842G>T. At 48 hours post-transfection, the culture media were replaced with fresh DMEM+10% FBS and cells were either treated with the protein biosynthesis inhibitor CHX at 50 µg/mL, the proteasome inhibitor MG132 at 10 µM or the lysosomal inhibitor bafilomycin A1 at 100 nM, incubated for various times, then lysed. Total cell lysates were analysed by western blotting as described above.

### Expression of glutathione S-transferase (GST) fusion protein and GST pull-down assay

The following primer pairs containing *Sma* I for forward primer and *Not* I for reverse primer were used to generate GST-tagged recombinant plasmids: wild-type *EPHA2* SAM domain, Forward primer 5′-tgttgcccgggattccgcacggtgtccgagtg-3′ and reverse primer 5′-ccttctcgagtcaagtgttcacctggtcctt-3′; and mutant *EPHA2* SAM domains, forward primer 5′-tgttgcccgggattccgcacggtgtccgagtg-3′ and reverse primer 5′-ccttctcgagtcagaaataaataaagtcccc-3′. PCRs were performed with the following cycle conditions: 95°C for 30 sec, 54°C for 30 sec, and 72°C for 30 sec for 25-cycles. PCR products were cloned into pGEX5X-1 expression vector. All GST-tagged SAM domain constructs were transformed into BL21 (DE3) *E. coli*, the proteins were induced with 1 mM isopropyl-β-D-thiogalactoside (IPTG) at 37°C for 4 hours. Whole-cell extracts were prepared and separated into soluble and insoluble fractions, and then the amount of recombinant GST fusion proteins were determined by coomassie or silver staining. For GST-wild-type SAM, GST-c.2819C>T SAM and GST-c.28442G>T SAM, 66 amino acids from the EphA2 SAM domain were fused to GST in frame at the 3′ end, resulting fusion proteins with the molecular weight of 33.6 kDa. For GST-c.2915_2916delTG SAM and GST-c.2826-G>A SAM, the mutated SAM domains contain 105 amino acids and 108 amino acids, respectively, resulting in molecular weights of 38 and 38.2 kDa.

### RNA preparation, reverse transcription (RT)-PCR and real-time PCR

To study the transcription of the *EPHA2* plasmids using semi-quantitative RT-PCR, total RNA was isolated from transfected cells using a QIAGEN total RNA isolation kit (Valencia, CA, U.S.A.). cDNA was generated by reverse transcription reaction using 5 µg of total RNA per sample with random primers and the SuperScript II Reverse Transcriptase kit (Invitrogen, Carlsbad, CA, USA). The following primer pairs were used for the analysis of *EPHA2* levels using PCR, generating a product of 248 bp: *EPHA2*, forward primer 5′-ttgtcatgtgggaggtgatg-3′ and reverse primer 5′-aaagtcagccagggtcttg-3′; and GAPDH, forward primer 5′-ttgccatcaatgaccccttca-3′ and reverse primer 5′-cgccccacttgattttgga-3′. PCRs were performed with the cycle conditions of 95°C for 30 sec, 60°C for 30 sec, and 68°C for 30 sec for 25 cycles.

Real-time PCR was performed using the ABI PRISM® 7000 sequence detection system and PCR reactions including SYBR Green dye. Results for target genes were normalized to glyceraldehyde-3-phosphate dehydrogenase (GAPDH) mRNA expression for each sample, and relative expression was calculated using the comparative threshold cycle method [72].

### Immunofluorescence staining

EphA2^−/−^ MEF (E13.5) cells expressing wild-type and mutant *EPHA2* genes were serum-starved overnight and then incubated with 2 µg/mL cross-linked ephrin-A5-Fc for 30 minutes. Cells were washed twice with phosphate buffered saline (PBS) at room temperature, and fixed in 4% paraformaldehyde (PFA) on ice for 30 minutes. Cells were permeablized with 0.3% PBS including Tween-20, and then blocked with 5% goat serum at room temperature for 2 hours. Secondary antibody Biotin-SP-AffiniPure goat anti-human IgG (Jackson Immuno-Research, Immuno-Research, West Grove, PA, USA) incubations were performed at room temperature for 1 hour. After extensive washing with PBS, CY3-conjugated streptavidin (Jackson Immuno-Research, Immuno-Research, West Grove, PA, USA) was added for 2 hours. Subsequently, samples were washed again with PBS and then incubated with anti-EphA2 antibody (1∶200, Abcam, Cambridge, MA), and images of stained cells were captured with a Nikon Eclipse C1 confocal microscope with 200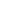 magnification.

### Cell migration assay

HEK293A cells were seeded on collagen-coated dishes and cultured for 24 hours. Cells expressing wild-type or mutant *EPHA2* constructs were serum-starved overnight. GFP-expressing vector, pEGFP-N1 was cotransfected with the *EPHA2* clones to identify transfected cells in the wound-healing assay. For ligand treatments, the culture media were replaced with fresh DMEM+10% FBS prior to stimulation. Cells were stimulated with 2 µg/mL cross-linked ephrin-A5-Fc. The migration distances of cells were monitored at the indicated time points (0, 24, and 48 hours) after wounding and quantified as described [73]. Images were captured using a Nikon Eclipse C1 confocal microscope with 40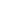 magnification. The relative migration distance of GFP-positive cells into wound was determined using NIH ImageJ and Adobe Photoshop CS3 software.

### Statistical analysis

Statistical analyses were performed using the Prism® software (GraphPad Software, La Jolla, CA). Statistical differences between multiple groups were analyzed using one-way analysis of variance (ANOVA). A two-tailed student t-test was used to analyze statistical significance between two groups. All values are presented as the standard deviation of the mean (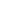
S.D.) from at least three independent experiments. Value of *P*<0.05 was considered to be statistically significant.

## Supporting Information

Figure S1 ***EPHA2*** **cataract mutations reduce SAM domain solubility in** ***E. coli*** **.** GST alone and GST-fusion proteins containing either wild-type SAM domain, two missense mutations c.2819C>T SAM and c.2842G>T SAM, the frameshift mutation c.2916_2916delTG SAM, and the splicing mutation c.2826-9G>A SAM were overexpressed in BL21 (DE3) *E. coli* with 1 mM IPTG at 37°C for 4 hours. (**A**) GST-fusion proteins were highly induced in bacterial cells by IPTG treatment. After induction with 1 mM IPTG, whole-cell extracts were prepared fractionated with SDS-PAGE, and stained with Coomassie Blue. (**B**) The solubility of the EPHA2 mutant proteins was significantly reduced. Whole-cell extracts were separated into soluble (S) and insoluble (I) fractions, and then the amount of soluble and insoluble recombinant GST fusion proteins were determined by silver staining. Arrowheads indicate the position of the fusion proteins.
(TIF)

Figure S2
**EPHA2 degradation is mediated by proteasomal pathway.** (**A**) MG132 prevents degradation of EPHA2 protein. HEK293T cells were treated for indicated time with the protein biosynthesis inhibitor CHX (50 µg/mL) and the proteasome inhibitor MG132 (10 µM). Cell lysates were immunoblotted with anti-EphA2 antibody. Lysates were resolved by SDS-PAGE and western blot analysis was performed using indicated antibodies as described in the Materials and Methods. The blot was reprobed with anti-α-tubulin as a loading control. (**B**) Graphs show EphA2 protein levels over time. Mean values are presented with 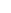
S.D as indicated.(TIF)

Figure S3 **Bafilomycin A1 does not stabilize EPHA2 protein.** HEK293T cells were transfected and treated for indicated time with the lysosomal inhibitor bafilomycin A1 (100 nM). Cell lysates were immunoblotted with anti-EphA2 antibody. Lysates were resolved by SDS-PAGE and western blot analysis was performed as described in the Materials and Methods. The blot was reprobed with anti-α-tubulin as a loading control.
(TIF)

Figure S4
**SAM domain of** ***EPHA2*** **is essential for ligand-independent promotion of cell migration of HEK293A cells.** (**A**) *EPHA2* SAM domain mutants lack migration promoting activity in HEK293A cells. HEK293A cells were grown to confluence and serum-starved for 24 hours. A scratch wound was made with a micropipette tip and the edge of cells as marked. 2 µg/mL cross-linked ephrin-A5-Fc was then added to the starvation media, and cells were allowed to migrate toward the center of the wound and photographed at the indicated times (representative figure of three independent experiments). The position of the initial scratch is indicated by dotted lines. Scale bar, 500 µm. (**B**) Quantification of *EPHA2* genes on HEK293A cell migration. The graphs represent the measurement of migration distance from three independent experiments. Mean values are presented with 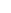
S.D as indicated. Statistical differences were analyzed using one-way analysis of variance (ANOVA) or calculated by a two-tailed student t-test. ***Black asterisks*** **,** comparison between time 0 and 24 hours and time 0 and 48 hours; ***Blue asterisks*** **,** comparison between the mock groups and the listed wild-type or mutant *EPHA2* genes at 24 or 48 hours; ***Red asterisks*** **,** comparison between untreated and treated conditions at 24 or 48 hours. ***, *P*<0.001; **, *P*<0.01; *, *P*<0.05; and ns, not significant. Values of *P*<0.05 were considered to be statistically significant.(TIF)

Figure S5
**Ligand-stimulated EPHA2 activation regulates Akt activation in αTN4-1 cells.** (**A**) Mutant EPHA2 genes have reduced ability to activate Akt. αTN4-1 cells were grown to near confluence, and growth factor-starved for 24 hours. 2 µg/mL cross-linked ephrin-A5-Fc was then added to the starvation media and cell lysates were immunoblotted with indicated antibodies. The blot was probed with anti-phospho-Akt (Ser473), and then reprobed with anti-α-tubulin as a loading control. (**B**) Wild-type and mutant *EPHA2* genes have similar activity in Akt activation when corrected for EPHA2 protein levels. Graphs show ratio of phosphor-Akt to total EPHA2. Quantification of phospho-Akt protein/total EPHA2 protein was determined using ImageJ software. Mean values are presented with 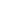
S.D as indicated. Statistical differences between multiple groups were analyzed using one-way analysis of variance (ANOVA). Values of *P*<0.05 were considered to be statistically significant. ns: No statistically significant difference between the two groups. Data for the other two mutants were not quantified, due to the very low levels of the signals.(TIF)
