# Supplementary figures and images for: Human Cataract Mutations in EPHA2 SAM Domain Alter Receptor Stability and Function
Source: PLoS One. 2012 May 3;7(5):e36564. doi: 10.1371/journal.pone.0036564 (PMC3343017; doi:10.1371/journal.pone.0036564)

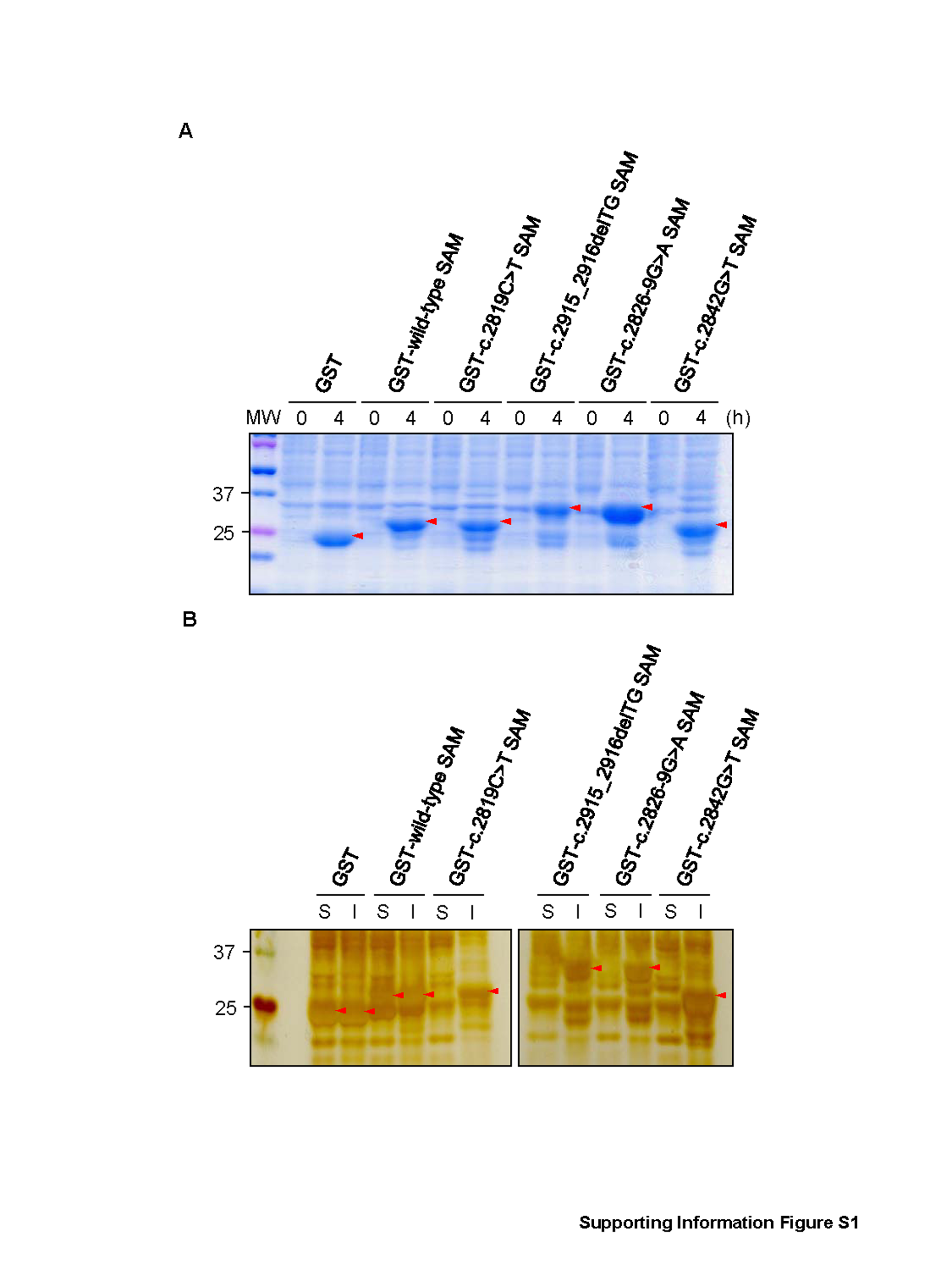

Supplement: Figure S1 — EPHA2 cataract mutations reduce SAM domain solubility in E. coli . GST alone and GST-fusion proteins containing either wild-type SAM domain, two missense mutations c.2819C>T SAM and c.2842G>T SAM, the frameshift mutation c.2916_2916delTG SAM, and the splicing mutation c.2826-9G>A SAM were overexpressed in BL21 (DE3) E. coli with 1 mM IPTG at 37°C for 4 hours. (A) GST-fusion proteins were highly induced in bacterial cells by IPTG treatment. After induction with 1 mM IPTG, whole-cell extracts were prepared fractionated with SDS-PAGE, and stained with Coomassie Blue. (B) The solubility of the EPHA2 mutant proteins was significantly reduced. Whole-cell extracts were separated into soluble (S) and insoluble (I) fractions, and then the amount of soluble and insoluble recombinant GST fusion proteins were determined by silver staining. Arrowheads indicate the position of the fusion proteins. (TIF) [file pone.0036564.s001.tif]

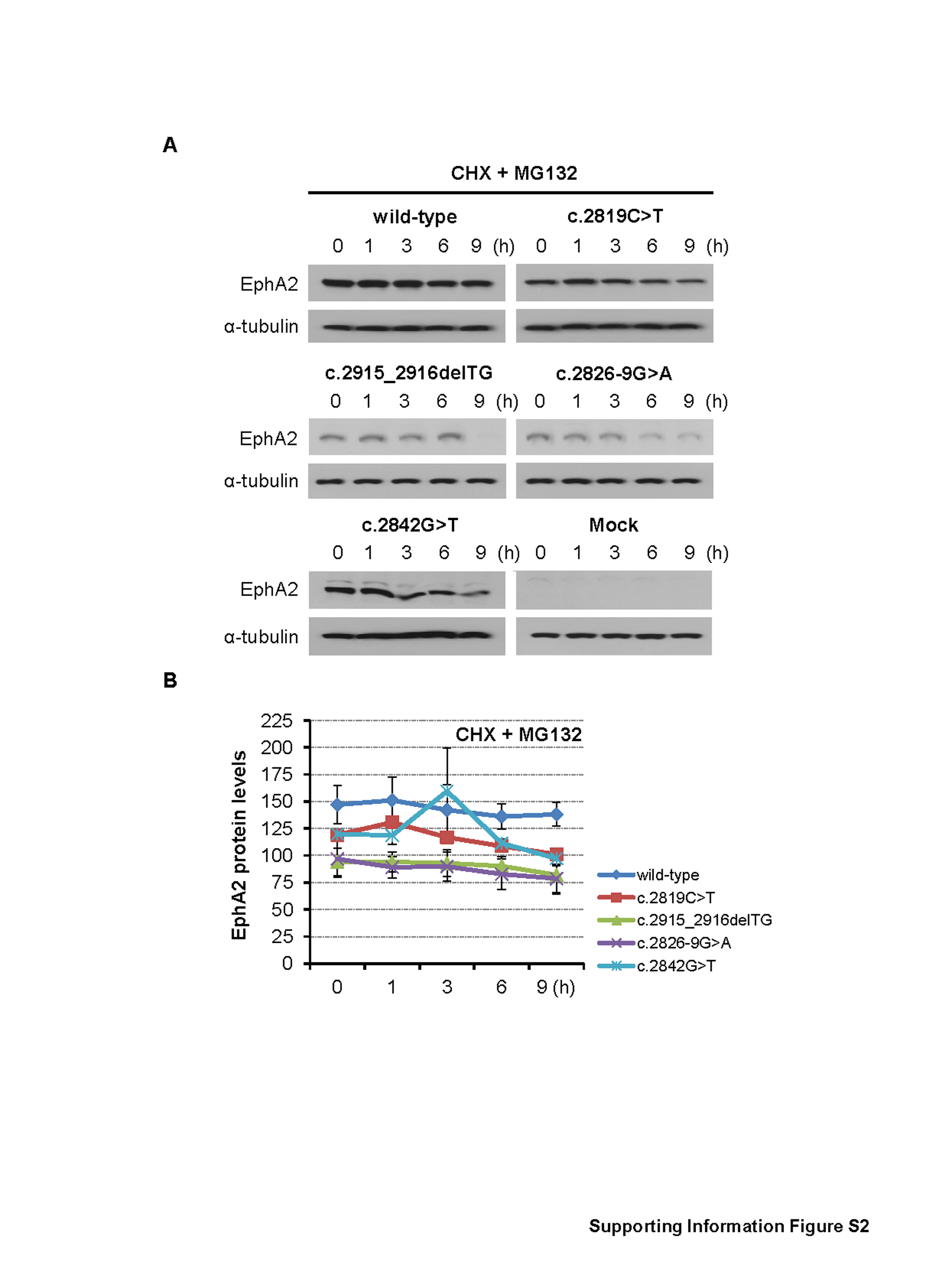

Supplement: Figure S2 — EPHA2 degradation is mediated by proteasomal pathway. (A) MG132 prevents degradation of EPHA2 protein. HEK293T cells were treated for indicated time with the protein biosynthesis inhibitor CHX (50 µg/mL) and the proteasome inhibitor MG132 (10 µM). Cell lysates were immunoblotted with anti-EphA2 antibody. Lysates were resolved by SDS-PAGE and western blot analysis was performed using indicated antibodies as described in the Materials and Methods. The blot was reprobed with anti-α-tubulin as a loading control. (B) Graphs show EphA2 protein levels over time. Mean values are presented with S.D as indicated. (TIF) [file pone.0036564.s002.tif]

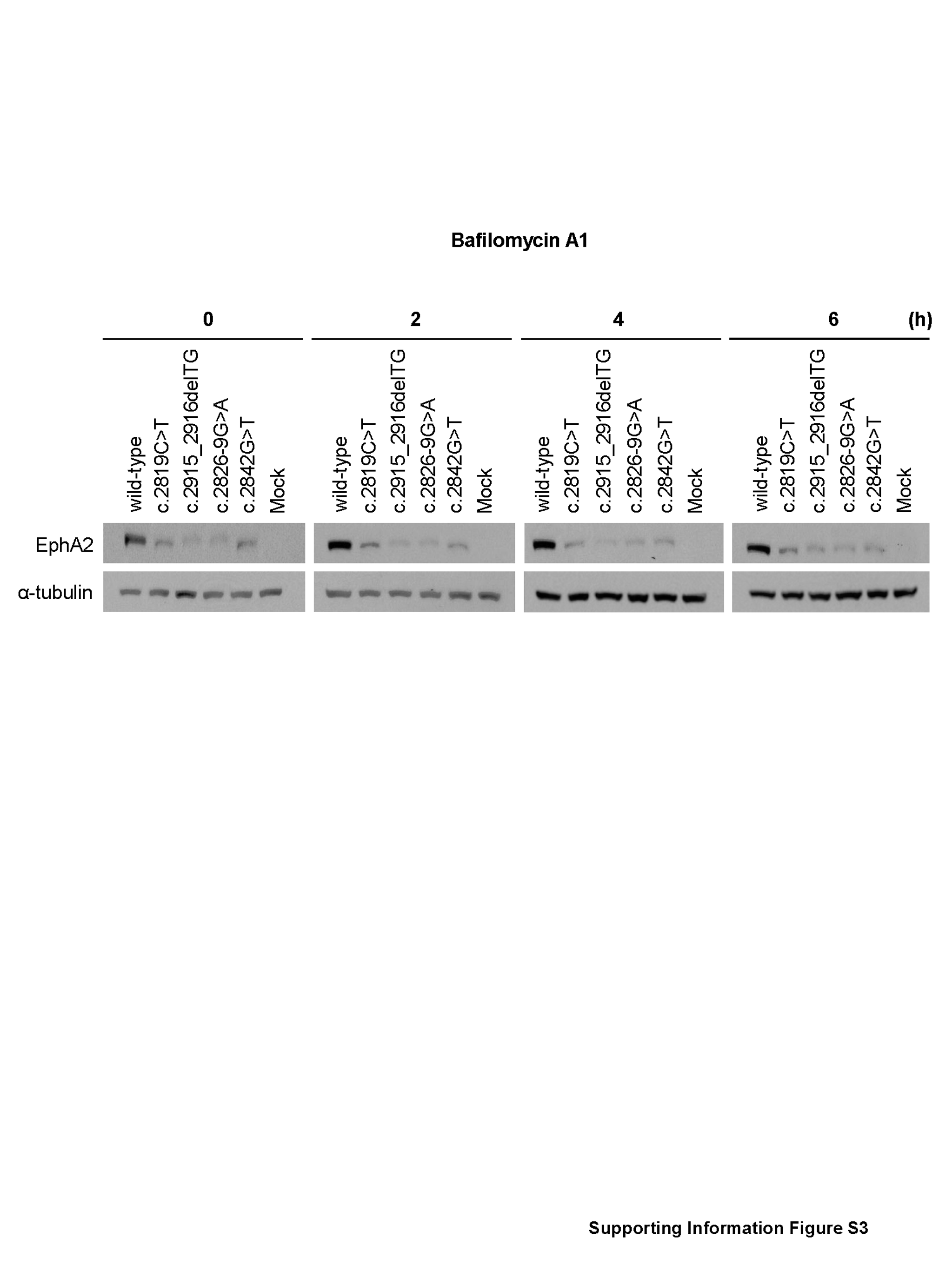

Supplement: Figure S3 — Bafilomycin A1 does not stabilize EPHA2 protein. HEK293T cells were transfected and treated for indicated time with the lysosomal inhibitor bafilomycin A1 (100 nM). Cell lysates were immunoblotted with anti-EphA2 antibody. Lysates were resolved by SDS-PAGE and western blot analysis was performed as described in the Materials and Methods. The blot was reprobed with anti-α-tubulin as a loading control. (TIF) [file pone.0036564.s003.tif]

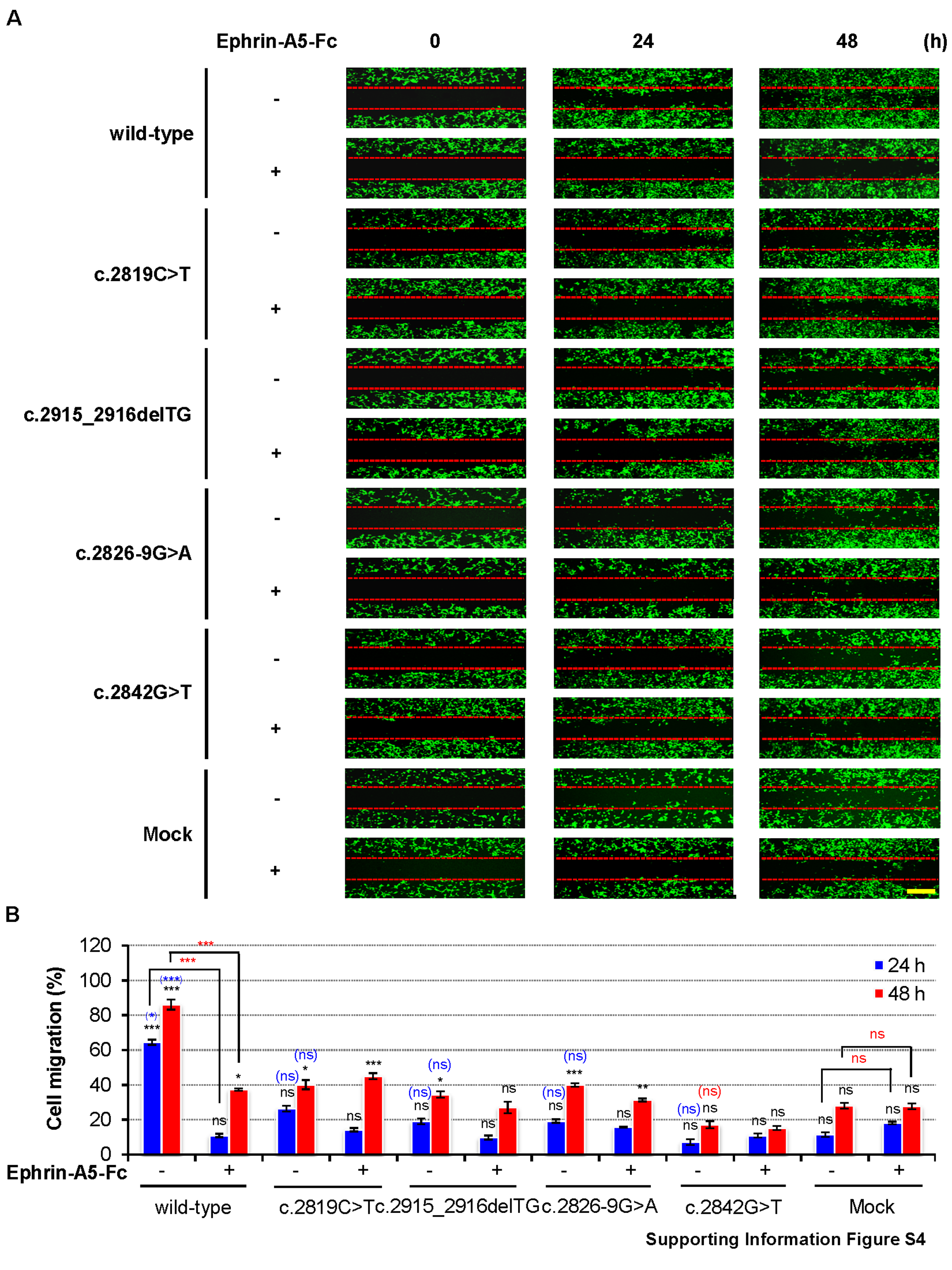

Supplement: Figure S4 — SAM domain of EPHA2 is essential for ligand-independent promotion of cell migration of HEK293A cells. (A) EPHA2 SAM domain mutants lack migration promoting activity in HEK293A cells. HEK293A cells were grown to confluence and serum-starved for 24 hours. A scratch wound was made with a micropipette tip and the edge of cells as marked. 2 µg/mL cross-linked ephrin-A5-Fc was then added to the starvation media, and cells were allowed to migrate toward the center of the wound and photographed at the indicated times (representative figure of three independent experiments). The position of the initial scratch is indicated by dotted lines. Scale bar, 500 µm. (B) Quantification of EPHA2 genes on HEK293A cell migration. The graphs represent the measurement of migration distance from three independent experiments. Mean values are presented with S.D as indicated. Statistical differences were analyzed using one-way analysis of variance (ANOVA) or calculated by a two-tailed student t-test. Black asterisks , comparison between time 0 and 24 hours and time 0 and 48 hours; Blue asterisks , comparison between the mock groups and the listed wild-type or mutant EPHA2 genes at 24 or 48 hours; Red asterisks , comparison between untreated and treated conditions at 24 or 48 hours. ***, P<0.001; **, P<0.01; *, P<0.05; and ns, not significant. Values of P<0.05 were considered to be statistically significant. (TIF) [file pone.0036564.s004.tif]

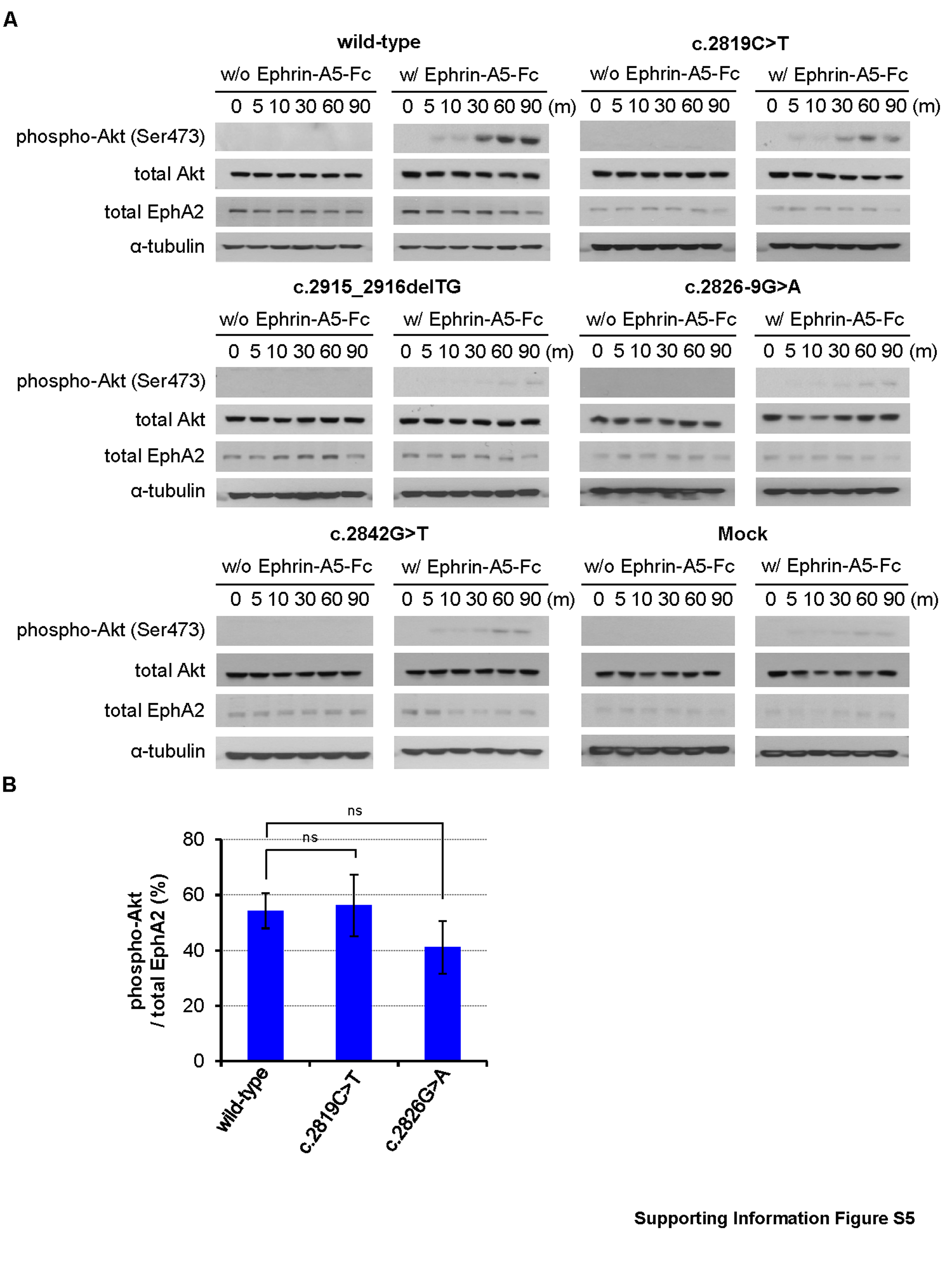

Supplement: Figure S5 — Ligand-stimulated EPHA2 activation regulates Akt activation in αTN4-1 cells. (A) Mutant EPHA2 genes have reduced ability to activate Akt. αTN4-1 cells were grown to near confluence, and growth factor-starved for 24 hours. 2 µg/mL cross-linked ephrin-A5-Fc was then added to the starvation media and cell lysates were immunoblotted with indicated antibodies. The blot was probed with anti-phospho-Akt (Ser473), and then reprobed with anti-α-tubulin as a loading control. (B) Wild-type and mutant EPHA2 genes have similar activity in Akt activation when corrected for EPHA2 protein levels. Graphs show ratio of phosphor-Akt to total EPHA2. Quantification of phospho-Akt protein/total EPHA2 protein was determined using ImageJ software. Mean values are presented with S.D as indicated. Statistical differences between multiple groups were analyzed using one-way analysis of variance (ANOVA). Values of P<0.05 were considered to be statistically significant. ns: No statistically significant difference between the two groups. Data for the other two mutants were not quantified, due to the very low levels of the signals. (TIF) [file pone.0036564.s005.tif]
